# Congenital Adrenal Hyperplasia: Diagnostic Pitfalls in Prolonged Neonatal Jaundice

**DOI:** 10.3390/clinpract11040102

**Published:** 2021-11-17

**Authors:** Nur Athirah Rosli, Md Yasin Mazapuspavina, Noor Shafina Mohd Nor

**Affiliations:** 1 Department of Primary Care Medicine, Faculty of Medicine, Universiti Teknologi MARA, Selayang Campus, Jalan Selayang Prima 7, Batu Caves 68100, Selangor, Malaysia; trahrosli@yahoo.com; 2 Department of Paediatrics, Faculty of Medicine, Universiti Teknologi MARA, Sungai Buloh Campus, Sungai Buloh 47000, Selangor, Malaysia; drshafina@uitm.edu.my; 3 Institute of Pathology, Laboratory and Forensic Medicine (I-PPerForM), Universiti Teknologi MARA, Sungai Buloh Campus, Sungai Buloh 47000, Selangor, Malaysia

**Keywords:** congenital adrenal hyperplasia, prolonged jaundice, hyperbilirubinemia, hyponatremia

## Abstract

Congenital Adrenal Hyperplasia (CAH) is a genetic disorder that leads to cortisol deficiency. However, prolonged neonatal jaundice is a rare presentation of CAH. The pathophysiology of hyperbilirubinemia in CAH is still ill-defined. Plausible causes are related to the synthesis of bile, maturity of the liver and adrenal function. This case reported a neonate who presented with severe prolonged jaundice that lasted for more than a month. A short Synacthen test confirmed diagnosis of CAH. He was started on steroid replacement. He had regular follow-up under paediatric endocrinologist and primary care physician for long-term monitoring and overall health care. This case demonstrates the importance of recognizing the clinical and biochemical features of CAH for early detection and referral. Long-term follow-up and monitoring is necessary due to the risk of complications and side effects of medications. This is the first case of CAH presented with persistent hyperbilirubinemia to be reported from Malaysia. The case describes the difficult workup that has been encountered in the patient’s care and management.

## 1. Introduction

CAH is a group of autosomal recessive disorders that results from enzyme deficiencies in the adrenal steroidogenesis pathway and affect the biosynthesis of mineralocorticoid and glucocorticoid, leading to low levels of aldosterone, cortisol and overproduction of androstenedione and subsequently high testosterone levels. Low cortisol levels cause a loss of negative feedback of ACTH secretion from the pituitary, which consequently increases ACTH secretion [1].

The majority of CAH cases (95%) are due to 21-hydroxylase deficiency (21OHD) and associated with pathogenic variants in the 21-hydroxylase (CYP21A2) gene. which is called classic CAH [2].

CAH with 21OHD is due to a mutation or deletion of either parent or both parents’ genes coding for the involved protein. Homozygous represents the abnormality of both genes, and heterozygous only affects one gene. If each of the genes are affected but differ in either mutation or deletion, this is called compound heterozygous [3]. Although there seem to be some exceptions, most heterozygous/carriers are asymptomatic.

Male infants with 46, XY genotype who are diagnosed with classic CAH have subtle genitalia hyperpigmentation but normal-appearing genitalia and/or an enlarged phallus. In these cases, there is a possibility of misdiagnosis and the infants may present with “salt-wasting”, which is the body’s inability to maintain normal salt and water homeostasis [4]. Most infants with classic CAH have a higher risk of mineralocorticoid deficiency and risk of adrenal crisis. Infants may present, at seven to 14 days of life, with symptoms of vomiting, diarrhoea, dehydration, hypotension, and hypovolemic shock, hyponatremia, hyperkalaemia, hypoglycaemia and metabolic acidosis. Some cases present with failure to thrive and, if presented late, at two to four years of age, with early pubarche, growth spurt, and adult body odour [5].

Female infants with 46, XX genotype who are diagnosed with classic CAH are typically born with ambiguous genitalia characterized by clitoral enlargement with or without hyperpigmentation, labial fusion and effects on the development of external genitalia. There is a possibility of profound virilisation, unrecognized genitalia and the case being assigned as male sex (with undescended testes) at birth in a 46, XX [6].

Patients may have several changes in glucocorticoid, mineralocorticoid, and sex steroid development that require hormone replacement therapy, depending on the form and severity of the steroid block. Presentations range from neonatal salt wasting and atypical genitalia to hirsutism and abnormal menstruation in adults. In many countries, the screening of neonates with high concentrations of 17-hydroxyprogesterone for classic (severe) 21OHD, the most common form of congenital adrenal hyperplasia, is in place, but ACTH stimulation testing may be necessary to confirm the diagnosis. ACTH test is also known as cosyntropin or the short Synacthen test [7].

Prolonged neonatal jaundice (PNNJ) is defined as visible jaundice with yellowish discoloration of the skin, mucous membrane and conjunctival icterus or serum bilirubin >85 μmol/L that persists beyond 14 days of life in a term baby and 21 days in a preterm baby [8].

The initial presentation of prolonged jaundice or persistent hyperbilirubinemia in CAH is not common. It has been shown that some hormonal disorders can alter liver function, particularly during the neonatal period. The pattern of liver dysfunction secondary to hormone deficiencies such as growth hormone deficiency, hypocortisolism or hypothyroidism is variable, including predominantly indirect hyperbilirubinemia, indirect hyperbilirubinemia that turns into cholestasis, cholestasis alone, or cholestasis with elevated hepatic enzymes [9].

It is postulated that hyperbilirubinemia in hypocortisolism results from diminished bile flow and/or excretion, which can be caused by affected intrahepatic bile production, defects in bile transmembrane transport or mechanical bile duct obstruction [10]. Most of the literature has shown that the common cause of prolonged jaundice is cholestasis. Fawwaz et al. defined cholestasis as an elevated serum direct bilirubin level (direct bilirubin levels >1.0 mg/dL or >17 μmol/L) [11]. However, the literature has also reported that the levels for suspicion of cholestasis for direct or conjugated bilirubin within the first 5 days of life could be as low as 5 μmol/L (0.3–0.4 mg/dL) [12,13,14]. Davis et al. suggested that, in the first 14 days after birth, the cut-off for elevated conjugated bilirubin may be greater than 0.5 mg/dL [15]. It is also defined as a defect in either the formation or excretion of bile, with a resulting increase in the serum or retained biliary components (bilirubin, bile acids, or cholesterol) [16].

To the best of our knowledge, this is the first case from Malaysia describing atypical presentation of prolonged neonatal jaundice in CAH. This also highlights the need to consider CAH as an important differential diagnosis of prolonged neonatal jaundice and in unexplained electrolyte abnormalities during the first few weeks of life.

## 2. Case Report

A term neonate had prolonged jaundice for 21 days starting from day 5 of life. He was born to a 31-year-old mother, para-3 via spontaneous vertex delivery at 37 weeks, after the induction of labour for oligohydramnios with amniotic fluid index (AFI) of 8.9. He was well, with a birth weight of 2660 g.

He was started on phototherapy for two days, when his serum total bilirubin was 365 μmol/L, with direct bilirubin of 12.1μmol/L at day-5 of life. He was put under phototherapy again, as his serum bilirubin was highest at day-16 of life (366 μmol/L) and was extensively investigated for severe prolonged unconjugated hyperbilirubinemia. There were no tea-coloured urine or pale coloured stools. Full blood picture and thyroid function tests were normal, excluding causes of haemolysis and hypothyroidism. Mother’s blood group is A+ and father is B+, with no consanguineous marriage.

He was then investigated for inborn error metabolism (IEM) and work-up for urine for reducing sugar, plasma amino acids, urine orotic acid. A galactosemia screening and blood spot for Galactose-1-Phosphate Uridyltransferase (GALT) were sent, which were reported as normal. The urine organic acid supported the non-diagnostic profile of IEM.

He had few episodes of rebound hyperbilirubinemia after discontinuation of phototherapy; thus, he was empirically started on phenobarbital. His bilirubin subsequently normalised and no rebound hyperbilirubinemia was noted after discontinuation of phenobarbital, thus ruling out the diagnosis of Crigler Najjar or Gilbert Syndrome.

He also was noted to have persistent hyponatremia, with levels ranging between 126 and 130 mmol/L with high normal potassium levels ranging between 4.3 and 5.6 mmol/L from day-16 up to three months of age, until the correct diagnosis was made and treated accordingly (Table 1). His urinalysis, serum osmolality, urine osmolality and urine pH were normal. His urine culture grew *Escherichia coli* and was treated with intravenous cefuroxime for a week. Despite sodium supplements commencing since day 16 of life, his sodium levels fluctuated and were noted to be significantly reduced during acute infection such as urinary tract infection (UTI) and pneumonia.

**Figure 1:**
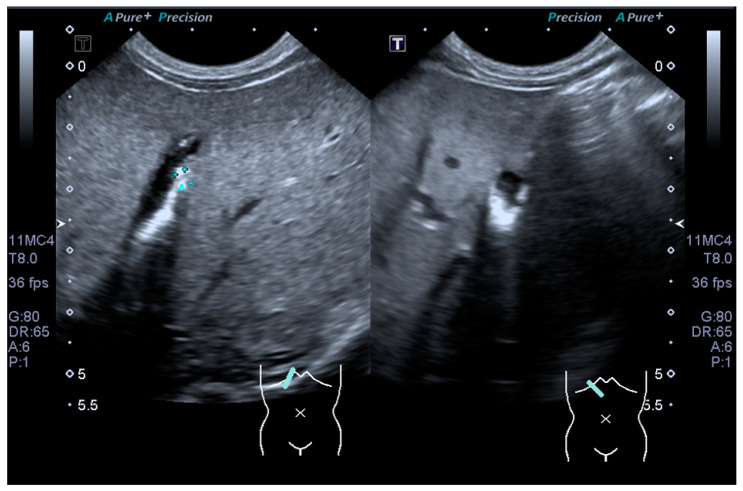
Ultrasound picture showing multiple echogenic foci within the gallbladder with posterior shadowing suggestive of sludge.

An ultrasound kidney-ureter-bladder (KUB) revealed bilateral hydronephrosis, right renal urinary tract dilatation (UTD) of moderate risk and left renal UTD of low risk. There were incidental findings of multiple echogenic foci within the gallbladder, with posterior shadowing suggestive of cholelithiasis (Figure 1). However, repeated ultrasound hepatobiliary by paediatric radiologist reported as gallbladder sludge.

During the hyponatremia periods, multiple differential diagnoses were outlined such as hyponatremia for investigation, recurrent UTI, Syndrome of Inappropriate Antidiuretic Hormone Secretion (SIADH) and persistent hyponatremia to rule out CAH. Further work-up showed normal levels of follicular stimulating hormone (FSH) and luteinizing hormone (LH); however, the serum renin and 17-OH progesterone (17OHP) levels were high, with levels of 241.1 nmol/L and >550 nmol/L, respectively. Subsequently, we proceeded with the short Synacthen test that confirmed the diagnosis of CAH.

**Figure 2:**
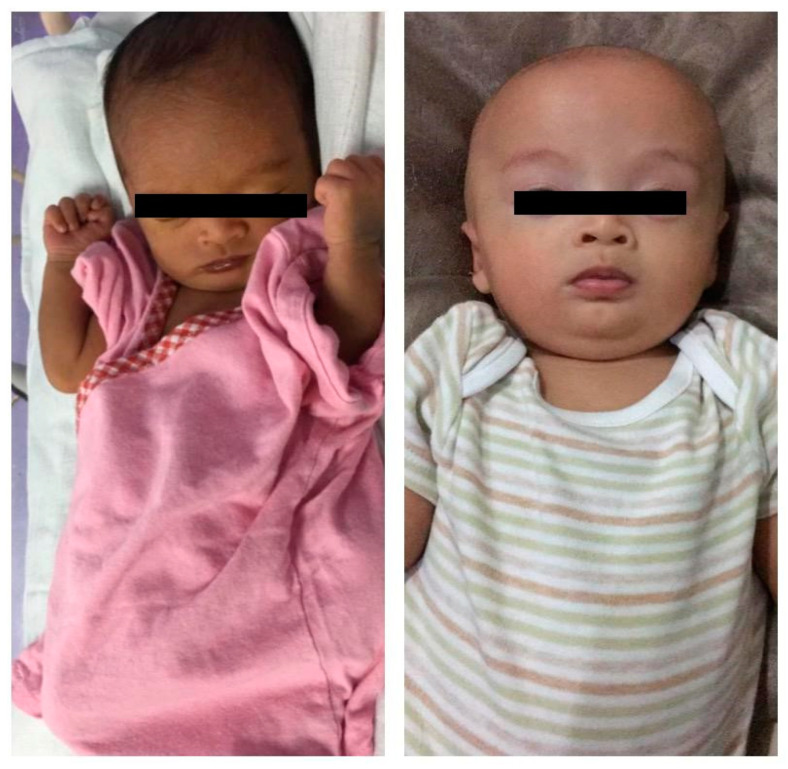
(**left**)—Day 14 of life, (**right**)—3-month-old (after treatment initiation).

He was then referred to a Paediatric Endocrinologist for further management and follow-up. Syrup hydrocortisone 1 mg eight hourly (10.3 mg/m^2^/day) and syrup fludrocortisone 50 mcg once daily, with supplementation of syrup sodium chloride (NaCl) 1 mL 12 hourly, commenced. The 17OHP consequently reduced in trend and the serum sodium levels normalised after seven months of treatment (Figure 2). Following that, a genetic test was conducted, which revealed that the patient was homozygous for the mutation in CYP21A2 that encodes the 21-hydroxylase enzyme, which is essential for adrenal steroidogenesis.

**Figure 3:**
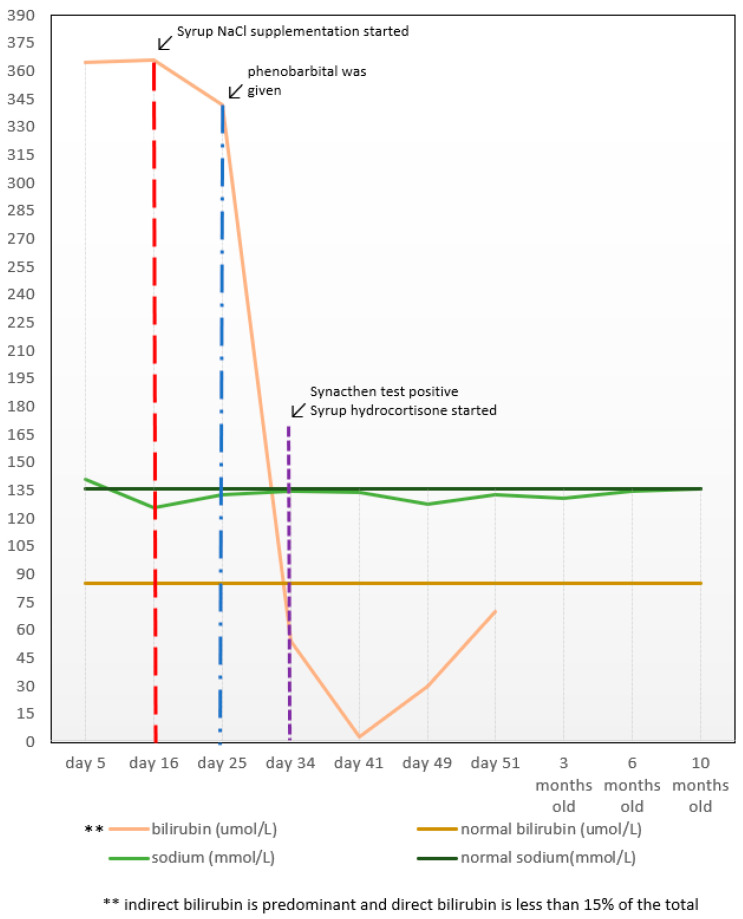
Clinical course of serum bilirubin and sodium levels of the patient.

At birth, he had a dark complexion (Figure 3) with a birth weight of 2.6 kg (25th centile); there were no signs of dysmorphism or chest wall abnormality. He has normal male genitalia, but was darkly pigmented and there were bilateral undescended testes. His left testis was not palpate and the right testis was palpable at the inguinal canal with a volume of 1 mL. There were no other abnormalities such as sacral pit, developmental dysplasia of hip or rocker bottom feet. At three months, he was small for his age, with both weight and height below the third centile. He had head lag, was unable to roll-over, or bear weight on legs and hands. At 10 months of age, his fine-motor, personal social skills and language improved and he caught up for his age, except for gross motor skills, as he still had head lag.

## 3. Discussion

CAH is a group of autosomal recessive disorders affecting cortisol and/or aldosterone biosynthesis depending on the spectrum of severity. The severe form is the classic type with obvious clinical phenotype. Classic CAH is subclassified as salt-losing or non-salt-losing (simple virilising), indicating the degree of aldosterone deficiency. 21OHD is the most common cause of classic CAH, occurring in approximately 95% of cases; this is characterised by cortisol deficiency, with or without aldosterone deficiency, and androgen excess. Non-classic CAH is usually asymptomatic or presents late, reflecting androgen excess rather than adrenal insufficiency [17].

In the Asian population, the incidence of CAH was about 1:44,000 in 2010 [18]. One study, conducted in 2015 in Malaysia, entitled “Clinical Presentation of Congenital Adrenal Hyperplasia in Selected Multi-ethnic Paediatric Population”, stated that 72% of neonatal males with CAH presented with salt wasting, 5% with virilising, 1% with ambiguous genitalia and hypertension, and 1% with ambiguous genitalia only [19].

PNNJ is not commonly reported as a presentation of CAH. A retrospective study conducted by Wijaya et al., on 362 infants with CAH, reported only 1.4% who presented with prolonged jaundice [20], while another study by Ozyilmaz et al., on 26 patients with CAH, reported that two of the patients presented with prolonged jaundice [21].

In PNNJ, the working diagnosis of CAH may not be considered during the differential diagnoses, as it can mimic other, more common hepatobiliary or gastrointestinal causes. Clinicians need to have a high index of suspicion, and specific investigations, including genetic testing, are to be performed when diagnosing CAH [22].

A few hormone abnormalities, particularly during the new-born period, have been shown to influence liver function. The pattern of liver dysfunction caused by hormone deficiencies including hypocortisolism can range from predominantly indirect hyperbilirubinemia to indirect hyperbilirubinemia that turns into cholestasis, cholestasis alone, or cholestasis with elevated hepatic enzymes [9].

One of the pathophysiologies that may suggest indirect hyperbilirubinemia that turns into cholestasis in CAH is a lack of glucocorticoids that leads to a decrease in bile secretion into the canaliculi. This is supported by animal model research, in which cortisol influences bile formation and bile flow is diminished in adrenalectomized rats [23]. Therefore, any disruption in either synthesis or secretion of bile, particularly in the immature infantile liver, could explain cholestasis [23]. However, the pathophysiological process remains unexplained. According to V. Theiler-Schwetz et al., an altered adrenal function is seen in rodent models of cholestasis, but the actual molecular component and the specific role for bile acids have yet to be defined. Possible molecular relationships between the bile acid and cortisol metabolism include their common precursor cholesterol as well as the bile acid receptors, which are also expressed in the adrenal glands [24]. The hepatoprotective systems in the neonatal liver are also relatively immature. The immaturity of these mechanisms may play a role in the pathogenesis of infantile cholestasis. There are now more reports of biliary sludge formation and gallstones in sick newborns, which could be caused by a combination of immature hepatic excretory activity and poor gallbladder function [25]. However, in this case, the objective measures to support cholestasis were not present as there were no results for bile acids and the profile of serum gamma-glutamyl-transferase (transpeptidase) (GGTP) activity.

To the best of our knowledge, this is the first case of CAH in Malaysia presenting with persistent hyperbilirubinemia. In this case, the patient had PNNJ with mild to moderate hyponatremia and high normal potassium, with the lowest sodium level of 126 mmol/L, and the highest potassium level of 5.4 mmol/L. This modest electrolyte imbalance might be the reason for the delayed diagnosis of CAH. The patient received phenobarbital as the treatment for hyperbilirubinemia, even though phenobarbital is an unproven therapy for cholestasis. Primarily, phenobarbital is used as an anticonvulsant. In addition, it only improved the hyperbilirubinemia, not the electrolyte imbalance. According to Stiehl et al., phenobarbital induces hepatic and microsomal enzymes to increase the conjugation and excretion of bilirubin [26].

In developing countries where newborn screening services are still unavailable, such as Malaysia, the diagnosis of CAH is sometimes missed. A high index of suspicion from the treating physician may help in the early identification and treatment of cases. A missed diagnosis of salt-losing CAH is associated with increased risk of early neonatal morbidity and mortality. In developed countries, with the advent of neonatal screening programs in assessing 17-OH progesterone by a rapid Delfia immunoassay, affected newborn males are typically diagnosed before they develop clinical symptoms [6]. On the contrary, if a newborn with CAH is not phenotypically identified or biochemical parameters are not screened, they will present with salt-wasting adrenal crisis at one to two weeks of age or with failure to thrive later. Thus, electrolyte disturbances in the presence of persistent hyperbilirubinemia should be considered as a possible congenital adrenal hyperplasia as the main pathology.

## 4. Conclusions

CAH may rarely present with persistent hyperbilirubinemia; thus, it is important for primary health care providers to consider a diagnosis of CAH as one of provisional diagnosis, particularly if it is associated with electrolyte imbalances. This high index of suspicion is crucial, as early recognition of CAH can lead to proper treatment, prevent severe electrolyte imbalances or salt-wasting crisis, and/or failure to thrive, thus reducing morbidity and complications.

## Figures and Tables

**Table 1:** Investigation results.

Date	Age	Total Bilirubin (μmol/L)	Direct Bilirubin (μmol/L)	Sodium (mmol/L)	17-OH P (nmol/L)	Cortisol (nmol/L)
23 May 2019	At birth	-	-	-	-	-
28 May 2019	Day 5	365	12.1	141	-	-
8 June 2019	Day 16	366	11.1	126	-	-
17 June 2019	Day 25	342	12.1	133	-	-
27 June 2019	Day 34	54	-	135	-	-
4 July 2019	Day 41	3.2	-	134	-	-
12 July 2019	Day 49	30	-	128	-	-
16 July 2019	Day 54	70	-	130	-	-
19 August 2019	Day 69	-	-	133	-	-
6 September 2019	3-month-old	-	-	131	241.1 (morning) >969.6 (30 min after) 916 (60 min after)	61 (morning) 76 (30 min after) 96 (60 min after)
20 December 2019	6-month-old	-	-	135	135.8	-
15 April 2020	10-month-old	7	-	136	46.7	-

## Data Availability

Data are available on request due to restrictions.

## References

[1] Parsa A., New M. (2016). Steroid 21-Hydroxylase Deficiency in Congenital Adrenal Hyperplasia. J. Steroid Biochem. Mol. Biol..

[2] Pignatelli D., Carvalho B.L., Palmeiro A., Barros A., Guerreiro S.G., Macut D. (2019). The Complexities in Genotyping of Congenital Adrenal Hyperplasia: 21-Hydroxylase Deficiency. Front. Endocrinol..

[3] Völkl T., Öhl L., Rauh M., Schöfl C., Doerr H. (2011). Adrenarche and Puberty in Children with Classic Congenital Adrenal Hyperplasia due to 21-Hydroxylase Deficiency. Horm. Res. Paediatr..

[4] White P.C., Speiser P.W. (2000). Congenital Adrenal Hyperplasia due to 21-Hydroxylase Deficiency. Endocr. Rev..

[5] Keil M.F., Bosmans C., Van Ryzin C., Merke D.P. (2010). Hypoglycemia during acute illness in children with classic congenital adrenal hyperplasia. J. Pediatr. Nurs..

[6] Speiser P.W., Arlt W., Auchus R.J., Baskin L.S., Conway G.S., Merke D.P., Meyer-Bahlburg H.F.L., Miller W.L., Murad M.H., Oberfield S.E. (2018). Congenital Adrenal Hyperplasia Due to Steroid 21-Hydroxylase Deficiency: An Endocrine Society Clinical Practice Guideline. J. Clin. Endocrinol. Metab..

[7] El-Maouche D., Arlt W., Merke D.P. (2017). Congenital adrenal hyperplasia. Lancet.

[8] Phak N.H., Thomas M.-I.H.-I. (2012). Paediatric Protocols for Malaysian Hospitals.

[9] Karnsakul W., Sawathiparnich P., Nimkarn S., Likitmaskul S., Santiprabhob J., Aanpreung P. (2007). Anterior pituitary hormone effects on hepatic functions in infants with congenital hypopituitarism. Ann. Hepatol..

[10] Khodadad A., Modaresi V., Kiani M.A., Rabani A., Pakseresht B. (2011). A case of lipoid congenital adrenal hyperplasia presenting with cholestasis. Iran. J. Pediatr..

[11] Fawaz R., Baumann U., Ekong U., Fischler B., Hadzic N., Mack C.L., McLin V.A., Molleston J.P., Neimark E., Ng V.L. (2017). Guideline for the Evaluation of Cholestatic Jaundice in Infants: Joint Recommendations of the North American Society for Pediatric Gastroenterology, Hepatology, and Nutrition and the European Society for Pediatric Gastroenterology, Hepatology, and Nutrition. J. Pediatr. Gastroenterol. Nutr..

[12] Harpavat S., Finegold M.J., Karpen S.J. (2011). Patients with biliary atresia have elevated direct/conjugated bilirubin levels shortly after birth. Pediatrics.

[13] Harpavat S., Garcia-Prats J.A., Shneider B.L. (2016). Newborn Bilirubin Screening for Biliary Atresia. N. Engl. J. Med..

[14] Harpavat S., Ramraj R., Finegold M.J., Brandt M.L., Hertel P.M., Fallon S.C., Shepherd R.W., Shneider B.L. (2016). Newborn Direct or Conjugated Bilirubin Measurements As a Potential Screen for Biliary Atresia. J. Pediatr. Gastroenterol. Nutr..

[15] Davis A.R., Rosenthal P., Escobar G.J., Newman T.B. (2011). Interpreting conjugated bilirubin levels in newborns. J. Pediatr..

[16] Weiss A.K., Vora P.V. (2018). Conjugated Hyperbilirubinemia in the Neonate and Young Infant. Pediatr. Emerg. Care.

[17] Merke D.P., Bornstein S.R. (2005). Congenital adrenal hyperplasia. Lancet.

[18] Trakakis E., Basios G., Trompoukis P., Labos G., Grammatikakis I., Kassanos D. (2010). An update to 21-hydroxylase deficient congenital adrenal hyperplasia. Gynecol. Endocrinol. Off. J. Int. Soc. Gynecol. Endocrinol..

[19] Thambiah S., Ahmad Z., Hambali Z., Osman M., Zain M., Zain F., Hong J. (2015). Clinical presentation of congenital adrenal hyperplasia in selected multiethnic paediatric population. Malays. J. Med. Health Sci..

[20] Wijaya M., Huamei M., Jun Z., Du M., Li Y., Chen Q., Chen H., Song G. (2019). Etiology of primary adrenal insufficiency in children: A 29-year single-center experience. J. Pediatr. Endocrinol. Metab..

[21] Özyilmaz B., Aydin M., Gönül O. (2018). Correlation of phenotype with the CYP21 gene mutation analysis of classic type congenital adrenal hyperplasia due to 21-hydroxylase deficiency. J. Exp. Clin. Med..

[22] Ali N., Zafar F., Bangash A., Malik A., Mohammedi K. (2014). Congenital adrenal hyperplasia with cholestatic jaundice. J. Pak. Med. Assoc..

[23] Bauman J.W., Chang B.S., Hall F.R. (1966). The effects of adrenalectomy and hypophysectomy on bile flow in the rat. Acta Endocrinol..

[24] Theiler-Schwetz V., Zaufel A., Schlager H., Obermayer-Pietsch B., Fickert P., Zollner G. (2019). Bile acids and glucocorticoid metabolism in health and disease. Biochim. Biophys. Acta (BBA)-Mol. Basis Dis..

[25] Emerick K.M., Whitington P.F. (2002). Molecular basis of neonatal cholestasis. Pediatr. Clin. N. Am..

[26] Stiehl A., Thaler M.M., Admirand W.H. (1972). The effects of phenobarbital on bile salts and bilirubin in patients with intrahepatic and extrahepatic cholestasis. N. Engl. J. Med..

